# The Hospital Pageant

**Published:** 1921-05-28

**Authors:** 


### THE HOSPITAL PAGEANT.

Co-operation in publicity clearly attracts the interest of many of the executive officers of the voluntary hospitals of London, if one may judge by the keenness of those who attended the meeting last week to consider the desirability of organising a hospital pageant, a scheme originally suggested in these columns. Half the Metropolitan hospitals sent delegates, and the teaching hospitals were, with few exceptions, represented.

As will be gathered from the brief report of the proceedings (to be found on the next page), the question in all its aspects was thoroughly discussed, and as a preliminary step the blessing of King Edward's Fund and the Sunday and Saturday Funds is to be sought. Then, no doubt, a small and strong executive committee will be able to take the scheme in hand and shape it into something definite and workable. At present the resolution is "that it is desirable to hold a Hospital Week, including a Pageant."

As we remarked in a previous article, the how, when, and where of the pageant are matters of detail, important enough in themselves, but not so important as the feat of bringing the hospitals together for common action in appeal, which at last is nearing achievement. It is to be hoped that not one of the Metropolitan hospitals will stand out of the effort, and the public will not be allowed to get the impression, as is the case in some other collective undertakings, that only a selection of charities are interested and will reap the benefit. The whole idea is advertising in a large and new way. All the hospitals are bound to benefit, and therefore in the matter of the expenses each institution should join, possibly—to take a practical proposal made at the meeting—on the basis of numbers of beds. It is most important that the combination of institutions should be complete, and the public effect of an announcement of this nature will, we are sure, be a distinct factor in the general success of the organisation.

